# A novel laparoscopic non-resective technique for the management of strangulated Richter's hernia

**DOI:** 10.1016/j.ijscr.2022.107335

**Published:** 2022-06-22

**Authors:** Marleny Carmona, Eduardo Smith Singares

**Affiliations:** a Elson S. Floyd College of Medicine, Washington State University, United States of America; b Division of Trauma & Emergency Surgical Services, Kadlec Medical Center, United States of America

**Keywords:** Richter's hernia, Partial enterocele, Laparoscopic non-resective technique, Enteroplication, Case report

## Abstract

**Introduction and importance:**

The incidence of Richter's hernias has risen in part due to the increment use of laparoscopic surgery. The standard technique to manage a strangulated Richter's hernia is bowel resection with anastomosis. Alternatively, invagination of the necrotic area in the enterocele maintains a clean surgical field and allows for the use of a mesh when closing the abdominal wall. In a sterile surgical field, the use of a prosthetic reinforcement has shown advantages, including low rates of long-term complications and reduced rates of hernia recurrence.

**Case presentation:**

A 35-year-old male presented with a strangulated Richter's hernia in a periumbilical abdominal wall defect. In the Operating Theatre, the necrotic segment was managed laparoscopically by plication with invagination allowing for abdominal wall reconstruction with a mesh.

**Clinical discussion and conclusion:**

We propose the laparoscopic repair of Richter's hernia with plication and invagination whenever feasible, therefore avoiding a bowel resection and maintaining a clean surgical field; which allows for use of prosthetic mesh.

## Highlights

• Richter hernias are becoming more common, mostly as a result of port-site closure defects after laparoscopic surgery
• Avoiding bowel resection will increase the likelihood of a successful long-term mesh implant and decrease hernia recurrences.
• Likewise, avoiding a bowel anastomosis will prevent additional contamination while simplifying the procedure.

## 1 Background

Richter's hernia is a type of breach of the abdominal wall, in which <50 % of the intestine's circumference protrudes through the defect and becomes necrotic. As minimal access surgery has demonstrated improved outcomes compared to their open counterparts, its use has risen dramatically [1]. This increase in the use of the laparoscopic approach, which creates multiple small defects in the abdominal wall, has led to a surge in the incidence of Richter's enterocele, and currently they amount to around 10 % of all the incarcerated hernias upon presentation [2]. The abdominal wall reconstruction technique depends on the wound classification. If a bowel resection occurs the field is considered contaminated and a prosthetic implant should be avoided; as retrospective studies have demonstrated an association between the use of mesh in a contaminated field with sepsis, local infections, and bowel obstruction [3]. In a clean surgical field, however, the use of a mesh has shown advantages compared to suture repair, including decreased incidence of long-term complications and reduced rates of hernia recurrence [4], [5], [6], [7], [8], [9]. Hence, avoiding bowel resection when repairing a Richter's hernia (which would allow for the use of a mesh) is desirable. For the preparation of this case report we followed the recommendations of the ICJME and the Equator Network (available at http://www.equator-network.org/).The formatting of the manuscript follows the surgical extension for the CARE statement (available at http://www.scareguideline.com/) [10].

## 2 Case presentation

**Fig. 1:**
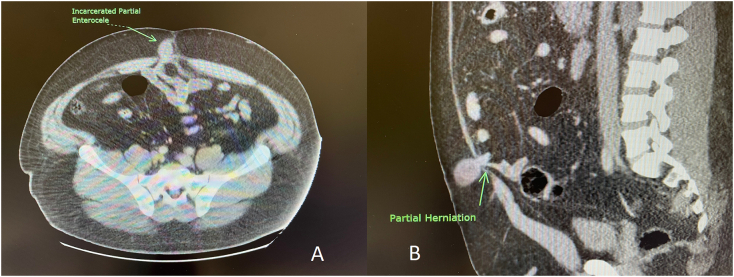
A and B: Computed Tomography scans of the abdomen (axial and sagittal cuts) demonstrating a hernia.

The patient is a 35-year-old male with a relevant past medical history of morbid obesity (with a BMI > 40) who presented initially to our free-standing Emergency Department with abdominal pain and a new periumbilical mass. He stated the pain that began while at work (he drives a tractor and lifts heavy bundles), had been colicky in nature and in-crescendo. He could not recall any one specific event or injury that resulted in this mass, and he first noticed it the day of presentation. Additional medical issues included hypertension and type-II diabetes. He had no previous surgical history and no prior diagnosis of ventral or umbilical hernia. On clinical exam his vitals were stable: his BP was 131/75, his pulse 85 BPM and his temperature was 36.7 °C. His respiratory rate was measured at 18 per minute and his pulse oxygenation recorded at 96 %. There was a non-reducible mass in the periumbilical area, exquisitely tender on palpation, which appeared mildly erythematous and warm. As part of his preoperative workup, he underwent blood work that showed a white blood cell count of 11.7 thousand per μL, platelets 301 thousand per μL, a hemoglobin of 17.5 G/dL. His chemistry was otherwise within normal limits except for elevated glucose (119 mg/dL), alkaline phosphatase (139 Units per liter) and Alanine transaminase (70 Units per liter). Hi serum lactate was measured at 1.0 mmol per liter, which is considered normal in our laboratory. His imaging included a computed tomography scan of the abdomen and pelvis with intravenous contrast (Fig. 1A-B), which showed a loop of small bowel contained in what appeared to be a hernia sac. The bowel content did not appear to involve the whole circumference of the small bowel loop. He was then transferred to our facility (approximately 10 h after the onset of the clinical picture), where our clinical exam confirmed the above findings. We scheduled the patient for emergency surgery.

**Fig. 2:**
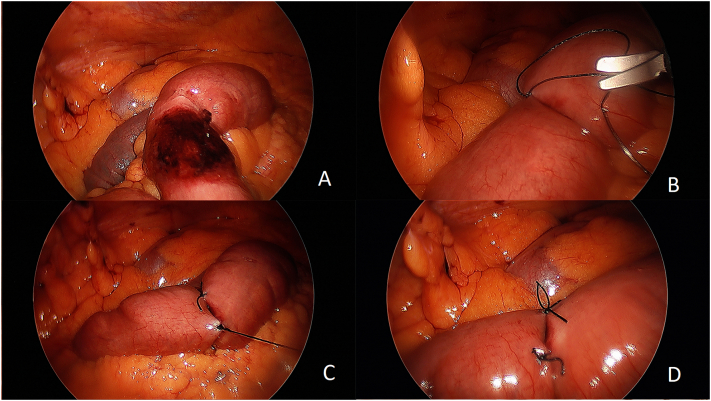
Intraoperative images of the Richter hernia and the process of its invagination. A) Ischemia of the previously incarcerated small bowel. B-D) The invagination process of the Richter hernia by plicating the surrounding healthy serosa of the bowel with multiple interrupted sutures (Lembert-style) of 3-0 silk.

The patient was taken to the operating room urgently for a diagnostic laparoscopy by the authors. The abdomen was entered at Palmer's point with a Verres needle. The loop of bowel that had been incarcerated was found spontaneously reduced, underneath the periumbilical hernia defect and appeared to be distal jejunum with a coin-like area of cyanotic tissue, amounting to <50 % of the total circumference of the affected loop of bowel, consistent with a Richter's hernia (Fig. 2A). The decoloration failed to resolve following the customary waiting period (10–20 min after reduction), and with the administration of 100 % oxygen. Indocyanine Green was not immediately available and therefore not administered (to confirm viability), however, the area in question lacked peristalsis and appeared softer and more pliant that the rest of the surrounding bowel; strongly suggesting established transmural necrosis. The Richter's strangulation was then managed by invagination, plicating the surrounding healthy serosa of the bowel with multiple interrupted sutures (Lembert-style) of 3-0 silk (Fig. 2B-D). The periumbilical hernia defect was measured to be 3 cm in diameter. It was managed with a 11.4 cm VentraLight circular mesh (Davol Inc., a subsidary of C. R. Bard, Inc. Warwick, RI), affixed in place with multiple fires of the 5 mm secure strap device (Ethicon Endosurgery, Cincinnati OH) using the double-crown technique. A total of three 5 mm laparoscopic ports were used for the operation.

The patient was discharged home on postoperative day two, tolerating a regular diet and with adequate pain control. Since his release, he has returned for follow up three times: at two weeks, five weeks, and ten weeks postop. No wound complications or hernia site issues were detected. There has been no clinical evidence for hernia recurrence up until his last follow-up.

## 3 Discussion

The entity we know today as Richter's hernia was first reported by Fabricius Hildanus in 1606, but it was August Gotlieb Richter that characterized the physiopathology of the condition as a partial enterocele; the protrusion of only part of the circumference of the intestine's antimesenteric border through a small defect of the abdominal wall [2], [10]. When compared to other types of hernia, Richter's progress more rapidly towards strangulation, but generates a clinical picture of intestinal obstruction significantly less often. The surgical management of strangulated Richter's hernia involves resection and re-anastomosis [2], [11] which results in a (potentially) contaminated field and necessarily precludes the use of mesh [4], [9].

After a careful search of all the available English biomedical literature in Pubmed, Medline and Google Scholar, we found that the first description of the invagination technique for the management of what we consider today to be Richter's hernias was reported as an open procedure by Joost M Horbach in 1986 [12] and reintroduced independently by Ahmed and Abo-elmagd in 2019 (also as an open procedure) [13]. It was presented as a safer technique in less experienced hands in more austere environments, and with outcomes than matched or surpassed those of more mainstream resective approaches [12], [13]. As far as we could surmise after our search, ours is the first description of this technique in conjunction with laparoscopic mesh hernioplasty. The advantages of this happy combination are clear: A laparoscopic suture plication is simpler (and cleaner) than an intracorporeal small bowel resection with re-anastomosis, resulting in no specimen removal and thus allowing for smaller port use. Technical pearls include the use of multiple interrupted sutures of non-absorbable material, applied at around 1 cm from the necrotic area (which ideally should encompass <50 % of the bowel circumference).

It is hypothesized that the necrotic bowel wall segment will slowly slough-off intraluminally, thus not compromising the lumen or creating a lead point for possible obstruction or intussusception. Obvious caveats for this hypothesis include the lack of long term follow up and the limited number of cases. As the use of minimally invasive approaches has shown clear benefits over open procedures (in terms of pain and recovery time), and prosthetic mesh hernioplasty in a clean surgical field has clear advantages (in terms of recurrence and overall complications) [4], [5], [6], [7], [8], [9]; we recommend the laparoscopic repair of Richter's hernias by bowel plication and invagination which maintains a clean surgical field and allows for the use of mesh.

## 4 Conclusion

There are limited options when confronted with strangulated bowel during the emergency management of a Richter's hernia. We advocate for the use of intestinal plication of the necrotic segment as a feasible alternative to bowel resection and anastomosis. It maintains a clean field and, more importantly, allows for mesh implantation, thus considered a superior hernia repair.

## 5 Patient perspective

The patient reported high satisfaction scores for this episode of care. During his last follow-up visit he confirmed that he has returned to work and had no pain left or need for refills on his pain prescription. He has not experienced any additional long-term issues such as change in bowel habits or other abdominal symptoms.

## List of abbreviations

BMI: body mass index
BP: blood pressure
BPM: beats per minute
cm: centimeters
G/dL: grams per deciliter
mg/dL: milligrams per deciliter
mm: millimeters
μL: microliter

## Ethical approval

The authors (MC and ESS) declare that this case report is exempted from ethics approval in the institution this case took place. As per existing policies and in accordance with current rules and regulations pertaining the protection of human subjects during research (including the Helsinki declaration) and applicable Federal Law pertaining the management of patient information, informed consent was obtained from the patient for surgical treatment, the acquisition of pictorial material and other elements related to his medical care and the possible use of this materials for teaching purposes, including publication on a scientific journal. The consent is available on the patient's paper chart and will be made available to the Editor upon request.

## Funding

Neither Kadlec Medical Center nor Washington State University identified and/or designated sources of funding for the realization of this project.

## Authors contribution

MC: (first author) performed the literature review, and revised the manuscript for style, language, and completeness.

ESS: (corresponding author) conceived the study and assisted with the literature review, collected the data for the case report and wrote the manuscript.

All authors have approved of the manuscript in its current (final form) before submission for consideration for publication.

## Guarantor

Eduardo Smith Singares.

## Research registration, availability of data and materials

All patient data and materials used for this manuscript are in store with the corresponding author and will be made available (unidentified) to requesting authorized parties upon request. The Case Report has been registered at www.researchregistry.com and the UIN is 7441 [available at https://www.researchregistry.com/browse-the-registry#home/registrationdetails/61b142135e81b2001efe9b5d/].

## Consent

Written informed consent was obtained from the patient for publication of this case report and accompanying images. A copy of the written consent is available for review by the Editorin-Chief of this journal on request.

## Declaration of competing interest

The authors (MC and ESS) declare that they have no conflict of interest pertaining to this case report.
